# A joint dataset of official COVID-19 reports and the governance, trade and competitiveness indicators of World Bank group platforms

**DOI:** 10.1016/j.dib.2020.105881

**Published:** 2020-06-19

**Authors:** Marcell Tamás Kurbucz

**Affiliations:** Department of Quantitative Methods, Faculty of Business and Economics, University of Pannonia, Egyetem utca 10., H-8200 Veszprém, Hungary

**Keywords:** COVID-19, Governance, Trade, Competitiveness, Data driven approach

## Abstract

The presented cross-sectional dataset can be employed to analyze the governmental, trade, and competitiveness relationships of official COVID-19 reports. It contains 18 COVID-19 variables generated based on the official reports of 138 countries (European Centre for Disease Prevention and Control, 2020 [1] and Beltekian et al. [2]), as well as an additional 2203 governance, trade, and competitiveness indicators from the World Bank Group *GovData360*(World Bank Group, 2020 [3]) and *TCdata360*(World Bank Group, 2020 [4]) platforms. From these platforms, only annual indicators from 2015 and later were collected, and their missing values were replaced with previous annual values, in descending order by year, until 2015. During preprocessing, indicators (columns) were filtered out when the ratio of missing values exceeded 50%. Then, the same filtration was applied for the ratio of missing values above 25% in the case of countries (rows). Finally, duplicated variables were removed from the dataset. As a result of these steps, the missing value rate of the employed indicators was reduced to 4.25% on average. In addition to the database, the Kendall rank correlation matrix is provided to facilitate subsequent analysis. The dataset and the correlation matrix can be updated and customized with an R Notebook file, which is also available publicly in Mendeley Data (Kurbucz, 2020 [5]).

**Specifications Table**

**Table:** 

**Subject**	Social Sciences
**Specific subject area**	The role of governmental, trade and competitiveness considerations in the formation of official COVID-19 data
**Type of data**	Tab separated text files (.txt) and a R Notebook file (.Rmd).
**How data were acquired**	Datasets are compiled in R.
**Data format**	Preprocessed and preanalyzed secondary data.
**Parameters for data collection**	2015 was the last year for which the values were taken into account during the collection of *GovData360* and *TCdata360* indicators and the replacement of their missing values. During the preprocessing, indicators were filtered out where the ratio of missing values exceeded 50%. Then, the same filtration was applied above 25% in the case of countries.
**Description of data collection**	To obtain the *GovData360* and *TCdata360* indicators, *data360r* (version: 1.0.8) R package [6] was used. Only annual indicators from 2015 and later were collected, and their missing values were replaced with previous annual values, in descending order by year, until 2015. During preprocessing, indicators (columns) were filtered out when the ratio of missing values exceeded 50%. Then, the same filtration was applied for the ratio of missing values above 25% in the case of countries (rows). Finally, duplicated variables were removed, and retained indicators were connected with 18 COVID-19 variables generated based on the official reports of 138 countries [1,2]. The Kendall rank correlation matrix was calculated based on the preprocessed dataset.
**Data source location**	Today's data on the geographic distribution of COVID-19 cases worldwide [1]: Author: European Centre for Disease Prevention and Control, URL: https://opendata.ecdc.europa.eu/covid19/casedistribution/csv, (accessed 25 May 2020). Data on COVID-19 (coronavirus) by Our World in Data [2]: Authors: D. Beltekian, D. Gavrilov, C. Giattino, J. Hasell, B. Macdonald, E. Mathieu, E. Ortiz-Ospina, H. Ritchie, M. Roser, URL: https://raw.githubusercontent.com/owid/covid-19-data/master/public/data/testing/covid-testing-all-observations.csv, (accessed 25 May 2020). World Bank Group *GovData360* platform [3]: Author: World Bank Group, URL: https://govdata360.worldbank.org/ (accessed 25 May 2020), Reached through: *data360r* (version: 1.0.8) R package [6]. World Bank Group *TCdata360* platform [4]: Author: World Bank Group, URL: https://tcdata360.worldbank.org/ (accessed 25 May 2020), Reached through: *data360r* (version: 1.0.8) R package [6].
**Data accessibility**	Repository name: Mendeley Data [5] Data identification number: DOI: 10.17632/hzdnxph8vg.3 Direct URL to data: http://dx.doi.org/10.17632/hzdnxph8vg.3

**Value of the data**

• This dataset can be employed to analyze the role of governmental, trade, and competitiveness considerations in the formation of official COVID-19 reports.
• Researchers in different fields of knowledge can use this dataset to investigate official COVID-19 data formation. The attached R Notebook might also be beneficial for policymakers and data scientists, not only to investigate pandemic reports but also to obtain a wide range of recent governmental, trade, and competitiveness indicators, in a preprocessed form.
• The provided dataset contains 18 COVID-19 variables, as well as 1102 governance and 1101 trade and competitiveness indicators. The large number of country features allows both data-driven and discipline-specific research. The preprocessed indicators of World Bank Group platforms can be used separately in various research fields (see, e.g., [7,8]).
• The Kendall rank correlation matrix is also provided to facilitate an in-depth analysis of the data.

## 1 Data description

The presented cross-sectional dataset can be employed to analyze the governmental, trade, and competitiveness relationships of official COVID-19 reports. It contains 18 COVID-19 variables generated based on the official reports of 138 countries [1,2], as well as an additional 2203 governance, trade, and competitiveness indicators from the World Bank Group *GovData360* [3] and *TCdata360* [4] platforms. Besides, the Kendall rank correlation matrix is provided to facilitate subsequent analysis. These datasets are complemented by the metadata of selected *GovData360* and *TCdata360* indicators, as well as country data that includes geographic coordinates, making it easier to visualize the results of subsequent analyses. These datasets can be generated in a contemporary form using the provided R Notebook. The current version was compiled on May 25, 2020. The complete list of uploaded files (including the raw data of figures and tables) is as follows.

**Datasets:**

a **Country data** *(country_data.txt*): Country data.
b **Metadata** *(metadata.txt)*: The metadata of selected *GovData360* and *TCdata360* indicators.
c **Joint dataset** *(joint_dataset.txt)*: The joint dataset of COVID-19 variables and preprocessed *GovData360* and *TCdata360* indicators.
d **Correlation matrix** *(correlation_matrix.txt)*: The Kendall rank correlation matrix of the joint dataset.

**R Notebook:**

• **Data generation** *(data_generation.Rmd)*: Datasets were generated with this R Notebook. It can be used to update datasets and customize the data generation process.

**Raw data of figures and tables:**

• **Raw data of** Fig. 2 *(raw_data_fig2.txt)*: The raw data of Fig. 2.
• **Raw data of** Fig. 3 *(raw_data_fig3.txt)*: The raw data of Fig. 3.
• **Raw data of** Table 1 *(raw_data_table1.txt)*: The raw data of Table 1.

**Table 1:** Variables description.

Variable ID	Type	Description	Missing	Source	Dataset
***iso3***	char	ISO3 country code.	0%	[6]	a, c
***iso2***	char	ISO2 country code.	0%	[6]	a
***capitalCity***	char	The capital city of the country.	0%	[6]	a
***geo.lat***	float	The latitude coordinates of the country's capital.	0%	[6]	a
***geo.lng***	float	The longitude coordinates of the country's capital.	0%	[6]	a
***population***	int	The population of the countries (2018).	0%	[1]	a
***id***	char	The ID of the indicator.	0%	[3,4,6]	b
***name***	char	The name of the indicator.	0%	[3,4,6]	b
***definition***	char	The definition of the indicator.	0%	[3,4,6]	b
***valueType***	char	The type of the indicator.	0%	[3,4,6]	b
***subindicatorType***	char	Type of the sub-indicator.	0%	[3,4,6]	b
***unit***	char	The unit of the indicator.	0%	[3,4,6]	b
***datasetId***	char	The ID of the dataset that contains the indicator.	0%	[3,4,6]	b
***dataset***	char	The name of the dataset that contains the indicator.	0%	[3,4,6]	b
***datasetLink***	char	The URL of the dataset that contains the indicator.	0%	[3,4,6]	b
***dyssincefstcase***	int	The number of days since the first case.	0%*	[1]	c, d
***dyssincefstdeath***	int	The number of days since the first death.	12.3%*	[1]	c, d
***dyssincefsttest***	int	The number of days since the first test.	42.8%*	[2]	c, d
***cases15dysaftfst***	int	The total number of cases after 15 days from the first case.	0.7%*	[1]	c, d
***deaths15dysaftfst***	int	The total number of deaths after 15 days from the first death.	14.5%*	[1]	c, d
***tests15dysaftfst***	int	The total number of tests after 15 days from the first test.	42.8%*	[2]	c, d
***cases30dysaftfst***	int	The total number of cases after 30 days from the first case.	1.4%*	[1]	c, d
***deaths30dysaftfst***	int	The total number of deaths after 30 days from the first death.	19.6%*	[1]	c, d
***tests30dysaftfst***	int	The total number of tests after 30 days from the first test.	44.2%*	[2]	c, d
***cases45dysaftfst***	int	The total number of cases after 45 days from the first case.	1.4%*	[1]	c, d
***deaths45dysaftfst***	int	The total number of deaths after 45 days from the first death.	22.5%*	[1]	c, d
***tests45dysaftfst***	int	The total number of tests after 45 days from the first test.	47.1%*	[2]	c, d
***cases60dysaftfst***	int	The total number of cases after 60 days from the first case.	5.1%*	[1]	c, d
***deaths60dysaftfst***	int	The total number of deaths after 60 days from the first death.	50.7%*	[1]	c, d
***tests60dysaftfst***	int	The total number of tests after 60 days from the first test.	55.1%*	[2]	c, d
***totcases***	int	The total number of cases.	0%*	[1]	c, d
***totdeaths***	int	The total number of deaths	0%*	[1]	c, d
***tottests***	int	The total number of tests.	42.8%*	[2]	c, d
**i*d_g1_,id_g2_,…,id_gn_***	int, float, boolean	The IDs of indicators obtained from *GovData360*.**	3.30%	[3,6]	c, d
***id_t1_,id_t2_,…,id_tn_***	int, float, boolean	The IDs of indicators obtained from *TCdata360*.**	5.22%	[4,6]	c, d

*These variables were generated by the author. Note that if the given number of days has not yet elapsed since the specified event, the value is missing. The R Notebook is used to update the dataset. **The complete list of *GovData360* and *TCdata360* indicators is contained by the metadata. For these variables, the averages of the ratio of missing values are indicated.
• **Raw data of** Table 2 *(raw_data_table2.txt)*: The raw data of Table 2.

**Table 2:** The steps of the data generation.

Step	Description	Remark
1	Installing packages and loading libraries	The program recognizes installed packages.
2	Setting parameters	Default settings: *lastyr* = 2005; *cmaxmissing* = 0.5; *rmaxmissing* = 0.25.*
3	Collecting *GovData360* indicators	With missing value imputation.
4	Collecting *TCdata360* indicators	With missing value imputation.
5	Collecting COVID-19 variables	
6	Generating new COVID-19 variables	
7	Compiling and preprocessing the joint dataset	
8	Compiling the correlation matrix	Kendall *τ_b_* correlation matrix is calculated.
9	Compiling the country dataset and metadata	
10	Writing datasets into TSV files	New files have the same name as uploaded ones.

*The data generation process can be customized with these parameters. *lastyr* marks the last year whose values were still taken into account when indicators were collected from the *GovData360* and *TCdata360* platforms and their missing values were replaced. During preprocessing, we filtered out those indicators for which the missing value ratio exceeds *cmaxmissing*. Then, the same filtration was applied above *rmaxmissing* in the case of countries.
• **Raw data of** Table 3 *(raw_data_table3.txt)*: The raw data of Table 3.

**Table 3:** Kendall rank correlation between COVID-19 variables.

*Variable*	*(1)*	*(2)*	*(3)*	*(4)*	*(5)*	*(6)*	*(7)*	*(8)*	*(9)*	*(10)*	*(11)*	*(12)*	*(13)*	*(14)*	*(15)*	*(16)*	*(17)*	*(18)*
***(1) dyssincefstcase***	1.00																	
***(2) cases15dysaftfst***	-0.19	1.00																
***(3) cases30dysaftfst***	-0.06	0.72	1.00															
***(4)cases45dysaftfst***	0.07	0.56	0.78	1.00														
***(5) cases60dysaftfst***	0.14	0.44	0.62	0.80	1.00													
***(6) dyssincefstdeath***	0.02	0.02	0.05	0.07	0.05	1.00												
***(7) deaths15dysaftfst***	-0.12	0.37	0.35	0.30	0.25	0.20	1.00											
***(8) deaths30dysaftfst***	-0.11	0.27	0.27	0.24	0.20	0.33	0.77	1.00										
***(9) deaths45dysaftfst***	-0.11	0.26	0.26	0.23	0.20	0.39	0.69	0.89	1.00									
***(10) deaths60dysaftfst***	-0.03	0.26	0.29	0.29	0.26	0.29	0.60	0.80	0.92	1.00								
***(11) dyssincefsttest***	0.25	-0.06	0.00	0.05	0.06	-0.14	0.00	0.02	0.01	0.07	1.00							
***(12) tests15dysaftfst***	0.04	0.28	0.25	0.27	0.31	0.07	0.11	0.00	0.02	−0.04	-0.41	1.00						
***(13) tests30dysaftfst***	0.07	0.31	0.31	0.35	0.38	0.05	0.15	0.04	0.05	0.00	-0.30	0.84	1.00					
***(14) tests45dysaftfst***	0.13	0.34	0.36	0.40	0.41	0.05	0.17	0.06	0.07	0.03	-0.17	0.72	0.85	1.00				
***(15) tests60dysaftfst***	0.13	0.31	0.35	0.43	0.47	0.10	0.20	0.12	0.13	0.08	-0.13	0.67	0.73	0.83	1.00			
***(16) totcase***	0.36	0.25	0.39	0.56	0.72	0.05	0.16	0.12	0.11	0.20	0.05	0.37	0.41	0.44	0.47	1.00		
***(17) totdeath***	0.35	0.17	0.35	0.49	0.60	0.08	0.10	0.12	0.14	0.25	0.12	0.21	0.25	0.25	0.29	0.72	1.00	
***(18) tottest***	0.19	0.30	0.35	0.44	0.51	0.00	0.22	0.15	0.13	0.19	0.14	0.38	0.50	0.63	0.76	0.55	0.36	1.00

**Fig. 1:**
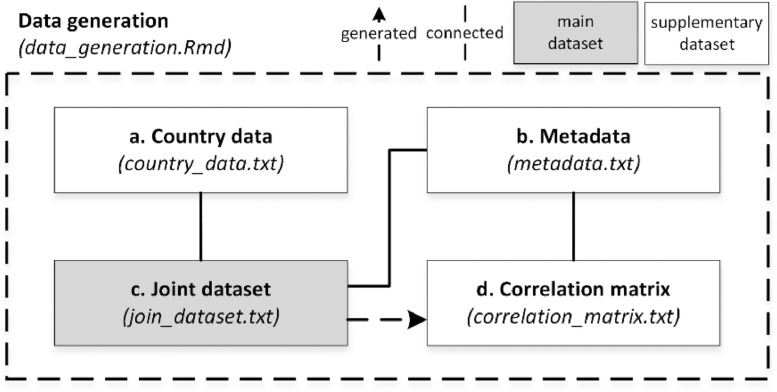
The relationship between uploaded files (Without raw data of figures and tables).

Fig. 1 illustrates the relationships between the R Notebook and datasets listed above.

**Fig. 2:**
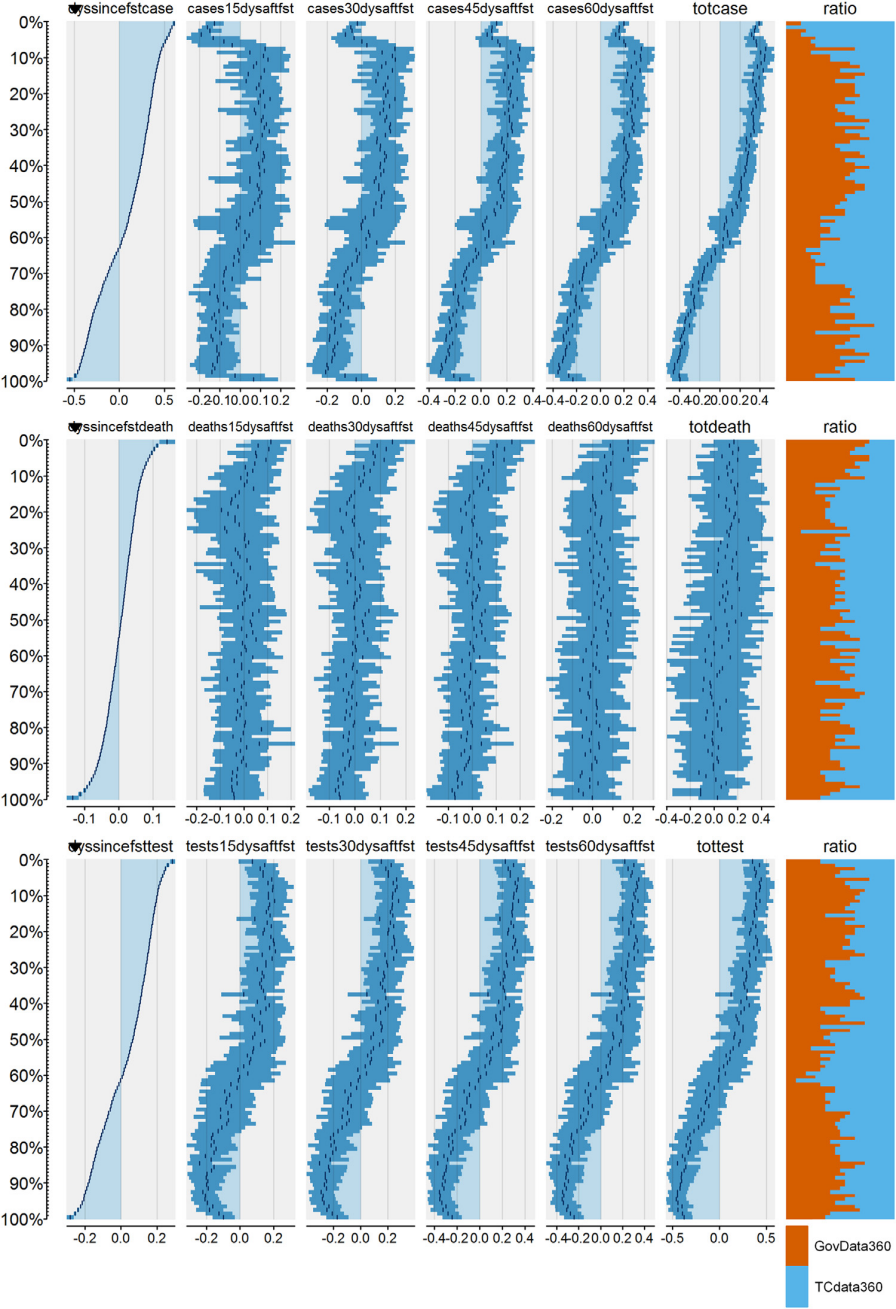
The relationship between the COVID-19, GovData360, and TCdata360 variables (COVID-19 variables (except for *dyssincefstcase, dyssincefstdeath*, and *dyssincefsttest*) are divided by population).

**Fig. 3:**
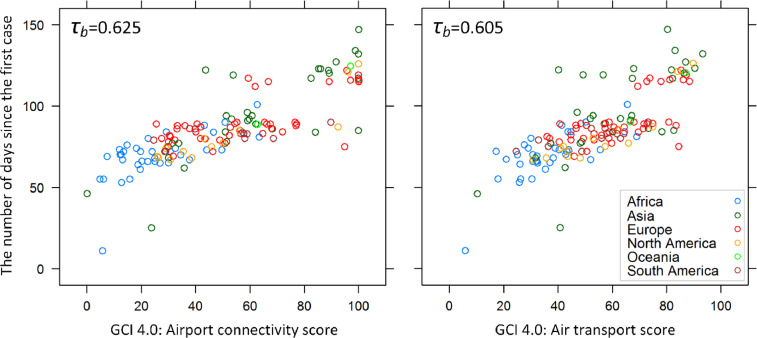
An example: Relationship of a COVID-19 variable to air transport indicators (For more information about GCI indicators, see metadata or [12]).

A detailed description of the extracted variables, their origin, the ratio of their missing values, and the ID of their datasets are shown in Table 1. Table 2 summarizes the generation process of these variables. Table 3, Figs. 2, and 3 relate to the Kendall rank correlation matrix. Table 3 includes the correlations between COVID-19 variables. Fig. 2 compares the connection of each COVID-19 variable with different governance, trade, and competitiveness indicators using table plots. Finally, Fig. 3 presents one of the many relationships contained by the correlation matrix that require further analysis. It illustrates the correlation between the air transport indicators of the Global Competitiveness Index (GCI) and the variable for the number of days since the first COVID-19 case.

## 2 Experimental design, materials and methods

To obtain the *GovData360* and *TCdata360* indicators, the *data360r* (version: 1.0.8) R package [6] was used. Only annual indicators from 2015 and later were collected, and their missing values were replaced with previous annual values, in descending order by year, until 2015. During preprocessing, indicators (columns) were filtered out when the ratio of missing values exceeded 50%. Then, the same filtration was applied for the ratio of missing values above 25% in the case of countries (rows). Finally, these data were connected with 18 COVID-19 variables. The Kendall rank correlation matrix was calculated using the preprocessed dataset and the *cor* function of the *stats* (version: 3.5.3) R package [9]. Before this calculation, COVID-19 variables (except for *dyssincefstcase, dyssincefstdeath*, and *dyssincefsttest*) were divided by the population of the respective countries, and the *use* argument of the *cor* function was set up to *pairwise.complete.obs* (for more information, see [10]). A detailed description of the extracted variables, their origin, the ratio of their missing values, and the ID of their datasets (see Fig. 1) are shown in Table 1.

### 2.1 Data generation

Datasets were generated in R. The process of data generation is summarized in Table 2.

### 2.2 Correlation matrix

In this subsection, the relationships between the variables are presented by using the Kendall rank correlation matrix. Table 3 contains the correlation matrix of COVID-19 variables.

To compare the relationship of each COVID-19 variable with different governance, trade, and competitiveness indicators, the *tabplot* (version: 1.3-4) R package [11] is used. *Tabplot* allows the exploration and analysis of large multivariate datasets with table plots. In our case, each column of this plot represents a COVID-19 variable, and each row represents a bin containing 100 indicators from *GovData360* and *TCdata360* platforms. Bars show the mean and the standard deviation of the correlations between the given COVID-19 variable and indicators contained in the bins. COVID-19 variables of cases, deaths, and tests are illustrated in different subplots. The last bar of these subplots displays the ratio of the *GovData360* and *TCdata360* indicators for each bin. For easier comparison, the correlation matrix is arranged in descending order of the first variable of the subplots (see Fig. 2).

The complete correlation matrix contains many relationships that require further analysis. Fig. 3 illustrates such a relationship between the air transport indicators of the Global Competitiveness Index (GCI) and the variable for the number of days since the first COVID-19 case.

## Declaration of Competing Interest

The author declares that he has no known competing financial interests or personal relationships which have, or could be perceived to have, influenced the work reported in this article.

## Appendix Supplementary materials

Image, application 1
